# Supplementary material for: Factors associated with treatment initiation delay among new adult pulmonary tuberculosis patients in Tigray, Northern Ethiopia
Source: PLoS One. 2020 Aug 21;15(8):e0235411. doi: 10.1371/journal.pone.0235411 (PMC7442238; doi:10.1371/journal.pone.0235411)
Supplement: S1 File — (DOCX) [file pone.0235411.s002.docx]

**Questionnaire to assessment of treatment initiation delay of TB and associated factors among new pulmonary TB patients in selected health facilities of Tigray, northern Ethiopia, 2019:**

**Instruction: Please fill the blank space for the open ended question and encircle the answer for the given choices of the study participants in the question item.**

| TB register no: |  | | | |
| --- | --- | --- | --- | --- |
| type of health facility (1. governmental ; 2. non-governmental): |  | | | |
| Name of the health facility |  | | | |
| Name of interviewer |  | | | |
| Date of the interview (dd/mm/yy): |  | | | |
| **Socio-demographic characteristics and risk factors:** |  | | | |
| TB patient code |  | | | |
| Age (years): |  | | | |
| Sex: |  | | | |
| Number of household members: |  | | | |
| Number of rooms in the house: |  | | | |
| Zone: -----------------------  Full address :-------------------------- |  | | | |
| Educational level: | - Illiterate/read & write - Primary - College and above | | | |
| Occupation: | - Employed - Farmer - House wife - Student - Daily labourer - Unemployed | | | |
| Income: | - In debt - o Income=expenses - Savings | | | |
| Residence: | o Urban  o Suburban  o Rural  o Homeless/displaced | | | |
| Marital Status: | o Married  o Single  o Divorced/separated  o Widowed | | | |
| Religion | - Orthodox - Muslim - Others | | | |
| Alcohol intake (do you drink alcohol?) | - Yes - No - If yes - Every day - Every other day - Not more than three days a week | | | |
| History of smoking: | o Never  o Current smoker  o Quitted smoking | | | |
| If smoker specify amount of daily consumption (number of cigarettes/day) |  | | | |
| Duration of smoking: | years--------; months---------- | | | |
| Previous exposure to TB patients: | 1.yes 2.no | | | |
| Other chronic diseases (eg. HIV/AIDS, Diabetes, COPD, Disability, etc): |  | | | |
| **History of current illness: Chief symptoms and date of onset of the illness:** | | | | |
| Cough | Date1 (dd/mm/yy): | | | |
| Fever | Date2 (dd/mm/yy): | | | |
| Loss of Weight: | Date3 (dd/mm/yy): | | | |
| Haemoptysis | Date4 (dd/mm/yy): | | | |
| Chest pain: | Date5 (dd/mm/yy): | | | |
| Others (specify) | Date6 (dd/mm/yy): | | | |
| Which symptom(s) made you seek healthcare: |  | | | |
| **Health seeking behaviour with onset of symptoms (before initial diagnosis)** | | | | |
| First action to symptom | Date(dd/mm/yy) | | | |
| HCP (Health Care Provider) |  | | | |
| Self medication |  | | | |
| Traditional medication |  | | | |
| Holy water |  | | | |
| Others (specify) |  | | | |
| Date first seen by HCP for the current illness (dd/mm/yy): |  | | | |
| Health facility of the HCP whom you first sought his consultation: | PHC  Public Hospital  Private Practice (Hospital or clinic)  Others please specify………………………. | | | |
| If private practice, specify the specialty of the HCP whom you first sought his consultation: | 1.Chest specialist  2. Internist  3. GP  4. Others (specify) | | | |
| Reasons of first consultation of the health facility (mentioned above) with the onset of symptoms | 0 Accessible  1 Confidence in getting cured  2 Services available anytime  3 Referred by previous health service  4 Free services  5 Advised by somebody  6 Others (specify) | | | |
| Reasons of non-consultation of health facility (coded 0,1in above) with the onset of symptoms (in case he did not consult HCP) (check): | 0 Too far  1 Too busy/long waiting time  2 Bad experience  3 Others (specify) | | | |
| Satisfaction with Care (score: 0 best, 3 worst please put the score in the provided space) | Are the health care providers supportive and respectful of people? |  | | |
|  | Before coming for diagnosis/ treatment, did you expect that the providers would be supportive and respectful to you? |  | | |
|  | Do most people in your community believe they will be treated supportively and respectfully by health care providers if they have TB? |  | | |
|  | Are you happy with the service you are getting from this facility? |  | | |
|  | Availability of services in PHC/TB centres |  | | |
|  | Prompt action from HCP in PHC |  | | |
|  | PHC well equipped |  | | |
|  | PHC giving free medicine |  | | |
|  | There is enough PHC in the area |  | | |
|  | Health facility workload |  | | |
|  | Waiting time (0:<= 15mn, 1: 15-30mn: 2: >30 mn-1 hr 3: >1 hr) |  | | |
| Perceived Causes of Delay in Health Seeking Behaviour: | 0 No delay  1 Fear of what would be found on diagnosis  2. Hoped their symptoms would go away on their own (denial and concealment)  3. Fear of social isolation  4. Economic constraints  5 Inadequate staff attitude  6 Poor quality of health services  7. Others (mention) | | | |
| TB stigma (Strongly agree=0 Agree=1 Average=2 Do not agree=3 Do not agree at all=4) | 1. Do you feel ashamed for having TB? | | |  |
|  | 2. Do you have to hide TB diagnosis from the  Other people? | | |  |
|  | 3. Does TB affect relation with the others? | | |  |
|  | 4. Is TB very costly due to long duration of the disease? | | |  |
|  | 5. Do you prefer to live isolated since you got TB diagnosis? | | |  |
|  | 6. Does the TB affect your work performance? | | |  |
|  | 7. Does TB affect marital relation? | | |  |
|  | 8. Does TB affect family responsibilities? | | |  |
|  | 9. Do you think there is less chances of marriage due to TB diagnosis? | | |  |
|  | 10. Does TB affect your family relations? | | |  |
|  | 11. Does TB cause female infertility? | | |  |
|  | 12. Does TB lead to serious complications during pregnancy? | | |  |
|  | 13. Does TB affect breast feeding? | | |  |
|  | 14. Does TB affect pregnancy outcome? | | |  |
|  | 15. Is a girl unable to decide for getting TB treatment? | | |  |
| Date of first TB diagnosis (dd/mm/yy): |  | | | |
| Number of health seeking encounters (HCP) before initial TB diagnosis: |  | | | |
| Action taken by HCP who made the initial TB diagnosis: [dd/mm/yy] | 1. Sputum examination date0:  2. X-ray date1:  3. GeneXpert date2:  4. Referral date3:  5. Others (specify) date4: | | | |
| X-ray: | 1.negative 2.positive 3.not performed | | | |
| Sputum smear | 1.Negative 2.positive: if positive grade a. +1; b. +2 c. +3 | | | |
| Date of initiation of treatment (dd/mm/yy): |  | | | |
| Accessibility to the public health facility providing treatment | Time to reach from home to the nearest public health facility:1. <1/2 hr ; 2. ½-1 hr; 3. > 1hr  Distance (in Km) from home to the nearest health facility providing treatment: | | | |
| Patient’s knowledge on TB | Have you previously heard of TB? Yes, no,  If yes Source of information on TB (select)  0 MOH campaign (media)  1 Education  2 Friends/relatives  3 TB disease in friends/relatives  4 Others (specify | | | |
| Correctness of information on TB  [Yes/right, 0] [No/wrong, 1] [not known, 2] | What kind of disease do you have? | |  | |
|  | Do you know what TB is? | |  | |
|  | Do you think that TB a serious disease? | |  | |
|  | Do you believe that TB is caused by mycobacterium TB? | |  | |
|  | Do you believe that TB transmitted by air? | |  | |
|  | Is TB hereditary? | |  | |
|  | Is TB contagious? | |  | |
|  | Is TB curable? | |  | |
|  | Do you know if there is a vaccine for TB? | |  | |
|  | Do you know the approximated duration of treatment? | |  | |
|  | Do you know that TB treatment is free? | |  | |
|  | Do you know the kind of TB drugs? | |  | |
