# Supplementary material for: Factors associated with treatment initiation delay among new adult pulmonary tuberculosis patients in Tigray, Northern Ethiopia
Source: PLoS One. 2020 Aug 21;15(8):e0235411. doi: 10.1371/journal.pone.0235411 (PMC7442238; doi:10.1371/journal.pone.0235411)
Supplement: Attachment — Submitted filename: response to reviewers.docx [file pone.0235411.s003.docx]

A rebuttal letter that responds to each point raised by the academic editor and reviewer(s)

Academic editor: Please include additional information regarding the survey or questionnaire used in the study and ensure that you have provided sufficient details that others could replicate the analyses. For instance, if you developed a questionnaire as part of this study and it is not under a copyright more restrictive than CC-BY, please include a copy, in both the original language and English, as Supporting Information.

Author response: thank you: I have provided the English version of the survey questionnaire and attached in the supporting information.

Academic editor: We suggest you thoroughly copyedit your manuscript for language usage, spelling, and grammar. If you do not know anyone who can help you do this, you may wish to consider employing a professional scientific editing service.  

Upon resubmission, please provide the following:

- The name of the colleague or the details of the professional service that edited your manuscript
- A copy of your manuscript showing your changes by either highlighting them or using track changes (uploaded as a *supporting information* file)
- A clean copy of the edited manuscript (uploaded as the new *manuscript* file)

Author response: thank you, I have sent the manuscript to professional Journal editor in Mekelle Univesity and I have attached the comments given as supporting information. The name of the reviewer is Dr. Afework Mulugeta who is an associate professor in Mekelle University and chief editor of the journals in the university. Dr. Afework is a visiting professor in many of Ethiopian universities. I had got his PhD from Oklahoma State University, USA.

Academic editor: PLOS requires an ORCID iD for the corresponding author in Editorial Manager on papers submitted after December 6th, 2016. Please ensure that you have an ORCID iD and that it is validated in Editorial Manager. To do this, go to ‘Update my Information’ (in the upper left-hand corner of the main menu), and click on the Fetch/Validate link next to the ORCID field. This will take you to the ORCID site and allow you to create a new iD or authenticate a pre-existing iD in Editorial Manager. Please see the following video for instructions on linking an ORCID iD to your Editorial Manager account: <https://www.youtube.com/watch?v=_xcclfuvtxQ>

Author response: thank you, I have got the ORCID ID.

Academic editor: Please include your tables as part of your main manuscript and remove the individual files. Please note that supplementary tables (should remain/ be uploaded) as separate "supporting information" files

Author response: thank you, I have included the tables in their appropriate place in the main manuscript.

5. Please include captions for your Supporting Information files at the end of your manuscript, and update any in-text citations to match accordingly. Please see our Supporting Information guidelines for more information: <http://journals.plos.org/plosone/s/supporting-information>.

Author response: Thank you I have included captions for the additional information as indicated below.

**Additional information**

questionnaire.docx: Questionnaire to assessment of treatment initiation delay of TB and associated factors among new pulmonary TB patients in selected health facilities of Tigray, northern Ethiopia, 2019; Languagereview.docx: Copyedit of the manuscript for language usage, spelling, and grammar.

Reviewer #1: 1. Better to short the topic. Its too long.

Author response: thank you for the constructive comment; I have tried to shorten the topic as the following: **Factors associated with treatment initiation delay among new adult pulmonary tuberculosis patients in Tigray, Northern Ethiopia**

2. data analysis and interpretation is in the satisfactory level.

Author response: thank you
3. conclusion is based on the results.

Author response: thank you
4. there is scientific value and it will add new data to the survey of TB

Author response: thank you

Reviewer #2: The article is interesting mainly because it describes as it's works the TB assistance in Northern Ethiopia. But it has some aspects needed to be corrected.
1: IT'S TOO LONG!.

Author response: thank you and I have tried to shorten the total size of the manuscript from its previous size of 24 into currently 20 pages including the tables.
A) Introduction: the last paragraph isn't correct.

Author response: Thank you very much for the constructive comment and I have tried to correct the paragraph as the following (available from line no: 56-65 page no: 2):

Almost all the studies in Ethiopia focused on either health centers or general hospitals (13, 15, 18) and lacks coverage across all of the levels of the health system but this research was done across all the levels of health system including health centers, general hospitals and referral hospitals. Most of the previous studies were also conducted when the directly observed treatment short course (DOTS) regimen was given for eight months which was later changed into six months starting from 2011 (19). Therefore, the present study was conducted to investigate delay to initiate treatment (patient and health system) and factors associated with delay among new PTB cases at health centers and hospitals in Tigray, Northern Ethiopia. . This would enable for the planning of specific interventions focusing patient, health system or both types of delays that yields the maximum benefit for TB patients and the community at large.

B) Methods and Materials: Very long. "Study setting" long, long...;

Author response: Thank you for the thoughtful comment and I have tried to shorten the methodology in general study setting in particular as the following (available from line no: 68-74 page no: 3):

This study was done in 16 health centers and 5 hospitals (4 general hospitals and one specialized referral hospital) from two zones of Tigray, Northern Ethiopia. Tigray Regional State is located at 12^o^15 - 4^o^57 longitude and 36^o^27- 39^o^59 latitude. Excluding Mekelle town, the state capital, there are six administrative zones in Tigray. The region has a total population of above 5.3 million which is about 6% of the total population of Ethiopia, over 80% live in the rural areas (20). There are 214 health centers and 38 hospitals (primary hospital, general hospital, and specialized hospitals) across the region (21).

C): Results: Tables numbers are bad located at the "profile of study participants";

Author response: Thank you; I have modified as the following: Summary results from bivariate and multivariable logistic regression showed that…… (Table 4) from line no:220-226 page no: 11 and (Table 5) from line no: 232-239 page no: 12

D: Discussion: too long;

Author response: Thank you and I have tried to make it short; currently I have reduced the number of pages from 4 in to 3 pages (line no: 244-323 and page no: 12-15).

E: Figures: ok;

Author response: Thank you

E: Tables: Table 1: the variable "Family size" it's not well located as a "Mean +- SD", can't to stay where it is.

Author response: Thank you and I have removed family size from the table.
